# Experimental Study on Upper-Limb Rehabilitation Training of Stroke Patients Based on Adaptive Task Level: A Preliminary Study

**DOI:** 10.1155/2019/2742595

**Published:** 2019-02-20

**Authors:** Lizheng Pan, Aiguo Song, Simei Wang, Suolin Duan

**Affiliations:** ^1^School of Mechanical Engineering, Changzhou University, Changzhou, China; ^2^Remote Measurement and Control Key Lab of Jiangsu Province, School of Instrument Science and Engineering, Southeast University, Nanjing, China; ^3^Medical Psychology, Changzhou Dean Hospital, Changzhou, China

## Abstract

During robot-aided motion rehabilitation training, inappropriate difficulty of the training task usually leads the subject becoming bored or frustrated; therefore, the difficulty of the training task has an important influence on the effectiveness of training. In this study, an adaptive task level strategy is proposed to intelligently serve the subject with a task of suitable difficulty. To make the training task attractive and continuously stimulate the patient's training enthusiasm, diverse training tasks based on grabbing game with visual feedback are developed. Meanwhile, to further enhance training awareness and inculcate a sense of urgency, a dynamic score feedback method is used in the design of the training tasks. Two types of experiments, functional and clinical rehabilitation experiments, were performed with a healthy adult and two recruited stroke patients, respectively. The experimental results suggest that the proposed adaptive task level strategy and dynamic score feedback method are effective strategies with respect to incentive function and rehabilitation efficacy.

## 1. Introduction

Stroke is a cerebral blood circulation disorder, which is mainly divided into hemorrhagic and ischemic stroke based on the pathogenesis [1]. Based on the report [1], the global prevalence of stroke was 42.4 million in 2015. In China, approximately 13 million individuals are stroke survivors, and the prevalence is increasing, with 2 million new stroke patients yearly [2]. Overall, the mean age of stroke patients worldwide is increasing, whereas the onset of stroke tends to be younger in China [2] and Sweden [3]. Due to the lack of effective treatment for the disease, stroke is characterized by high mortality and disability. How to effectively treat stroke and reduce the poor consequences is a common problem in the medical field.

Stroke is referred to as a cerebrovascular accident with a sudden decrease in blood supply to the brain tissue, which may result in brain tissue ischemia and brain cell damage. When the brain nerve cells are damaged, the body functions controlled by these nerve cells are impaired. Stroke treatment and rehabilitation are usually divided into two stages, namely, acute and chronic phases of stroke [4, 5]. During the acute phase, the patient's corresponding function is restored when the impaired neural connections are recovered within the called sensitive time-limited window. Based on neuroplasticity and compensation of brain function theory, in recent decades, many advanced stroke rehabilitation techniques have been developed and utilized, such as robot-aided device [6], virtual reality [7], brain stimulation, and constraint-induced therapy, for the patients in the chronic phase of stroke [8, 9]. To aid the patient in recovering the lost function to the greatest extent, these advanced techniques using unconventional drug therapy for the recovery of the patient's body functions have received increasing attention from researchers for the past few years.

Stroke may be associated with disabilities for the survivor. The disabilities usually affect the activities of daily living, such as motion ability, walking, speech, and cognition [10, 11]. In clinics, motor deficits are some of the most prevalent symptoms, and 69% of stroke patients have some degree of motion disability of the upper extremity [12]. Fortunately, clinic investigations in both human and animal models demonstrate that massive and intensive motion training can induce cortical changes and reorganization, which construct a relative ability to produce skilled action [4]. Thus, motor function improvements beyond the subacute stage might be induced by rehabilitative therapies. Exercise therapy plays an important role in functional recovery and reconstruction, and it is a popular therapeutic method for stroke rehabilitation. Effectiveness of motion training for motor function improvement has been widely reported [13]. In clinics, motion training is usually conducted by a physiotherapist. The traditional hand-to-hand treatment by a physiotherapist has many disadvantages, such as high labor intensity, low efficiency, and rehabilitation effectiveness varying with the physician. To effectively offer stroke patients with modern technology, all kinds of motion training robots are developed to replace the physiotherapist to offer the patient with designed motion training, which presents the advantages with recording process data, high convenience providing task-oriented practice, and high accuracy in measuring outcomes. In recent years, the rehabilitation robot has become a hot topic in the field of robotics. Robotics is increasingly used in poststroke upper extremity rehabilitation [14]. With regard to the upper extremity rehabilitation robot, studies have greatly contributed to the system mechanism design [15, 16], control method [17, 18], rehabilitation training method [19], visual feedback, and so on [20]. The aim of developing motion-rehabilitation training robots is to help patients affected with motor disability relearn motion skills based on the experience-dependent neural plasticity with robot-aided motion training. How to stimulate the enthusiasm of the subject to the greatest extent is one of the main considered issues throughout the design of the rehabilitation system. Many training or controlling strategies have been adopted to improve training motivation, such as varied training tasks, vivid visual feedback or virtual reality, friendly interaction, and intelligent control methods [21–23]. However, the training task is usually appointed in advance during the robot-aided motion exercise. Too difficult training tasks will lead the trainer to lose confidence, and too easy training tasks will lead to boredom. Therefore, the level of training tasks during one training session needs to be adjusted based on the training performances. Motion training with matching difficulty level can effectively stimulate the enthusiasm of the subject and make the movement undergo better cooperation.

In this study, the training task based on a game with various levels of difficulty was designed to improve the effectiveness of the rehabilitation training for robot-aided free movement training. Moreover, an adaptive strategy for selection of task level and dynamic visual feedback method were adopted; these interventions can provide the patients with the appropriate training task and motivational visual feedback, which may motivate interest in training and participation awareness.

## 2. Materials and Methods

### 2.1. Motion Training Type

In clinics, the motion training type of robot-aided rehabilitation exercise is usually varied with the rehabilitant stage and the state of illness. The motion training types are divided into three modes based on the condition that the robot provides auxiliary force: the passive, aided active, and free motions.

The passive motion training is usually utilized in the early recovery phase where the stroke patient does not present any motion ability, and the movement is fully towed by robot following the predefined trajectory. When the stroke patient possesses a certain active ability but cannot completely overcome gravity, the aided active motion is utilized to arouse the active movement consciousness. During the aided active motion, an appropriate aided force is supplied by the robot to help the subject perform training tasks based on the designed control algorithm. Free motion is used in the stage where the subject can fully overcome gravity. Free movement refers to the movement initiated by the patient himself, and the whole movement process is completely self-initiated by the patient. The end of robot manipulation follows the subject's hand and does not provide any force or any direction guidance for the movement of the patient. Free motion is fully controlled by the trainer, and the exercise process is actually a coordinated control process of body. The active participation of patients is conducive to accelerating the control of the central nervous system reconstruction on the affected limb. Meanwhile, subjects can freely move according to their wishes, which largely increase their confidence and inspire their motion enthusiasm.

With regard to each motion training type, the training form and control strategy are usually different due to the specific characteristics and goals. To increase interest in training, visual feedback techniques are usually used to design and develop free motion training. This study focuses on free movement training and evaluates the effective training methods using an adaptive training strategy and dynamic visual feedback.

### 2.2. Rehabilitation System Set-Up

**Figure 1:**
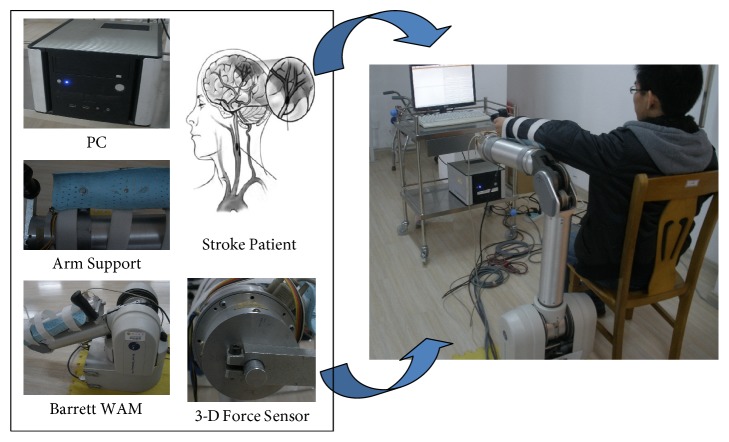
Motion rehabilitation system.

The constructed motion rehabilitation system mainly includes two parts: the hardware and software sections. The four-degree-of-freedom Barrett Whole Arm Manipulator (WAM), which has been widely used as an experimental platform in the medical field, was used as the main platform to construct the upper-limb motion rehabilitation system. The Barrett WAM can be well controlled in joint space, and each joint can be driven by setting the control torque; the position of each rotary joint is measured timely. Additionally, the Barrett WAM was designed with cable-driven technology, presenting outstanding back drivability and safety, which is suitable for an ideal hardware platform for motion training. To monitor the interactive force during rehabilitation, a three-dimensional (3-D) force sensor was developed and installed on the end-effector. An arm-support device was designed and installed to support the impaired limb for stroke patients to perform certain types of motion training. In this investigation, the motion rehabilitation system, which is presented in Figure 1, mainly consists of the WAM manipulator, 3-D force sensor, arm-support device, and controlling PC. More detailed information on the constructed motion rehabilitation system can be acquired from our previous studies [24, 25].

The software of the motion rehabilitation system works with Linux system running in the extern PC. During designing of the controlling software, real-time module Xenomai and multithread task management mechanism are used to improve instantaneity.

### 2.3. Training Task for Free Motion

Similar to what the patient stated to Reinkensmeyer [20] in a clinical trial, “If I cannot do it once, succeed at one time, why do it a hundred times?” Clinical studies have shown that the more active the patient, the better the outcome. To improve the efficacy of motion training, the designed training task should be attractive and can continuously stimulate the patient's training enthusiasm.

During spontaneous free movement training of the subject, if the goal set is unclear, but the patient is allowed to move at will, the training will be boring, and the patient will be easily tired. Therefore, to offer patients with lively and interesting rehabilitation training and effectively increase the patient's interest in training, the training task is designed with game combining visual feedback.

The upper limbs are mainly engaged in grasping movements in daily life. Considering the patient's desire to return to daily life and grasping the “object,” the game is presented in the form of grabbing the target. A certain number of targets are displayed on the screen, and the patient controls the position of the robot end-effector by free movement; visual feedback for this movement is provided with the movement of a virtual “hand” grabbing the object on the computer screen. To meet the needs of different levels of motion training, the game is set with three levels, and the difficulty level is distinguished by the number of targets and the size of the target icon. “Easy level” involves fewer targets and larger target icons; “hard level” involves a greater number of targets, and the target icons are smaller; and “medium level” presents with target icons of different sizes. To avoid the limitation of the monotony of the target scene, a rich scene mode was also designed in this study. The rich scene presents with some common goals of daily life, which aids the patient in thinking of the relevant actions performed previously in life and increases the awareness of the movement. For example, when the subject sees a lot of money on the screen, he instinctively tries to grab the money. The system can allow certain gravity compensation for the subject to perform free movement training according to his own physical condition.

**Figure 2:**
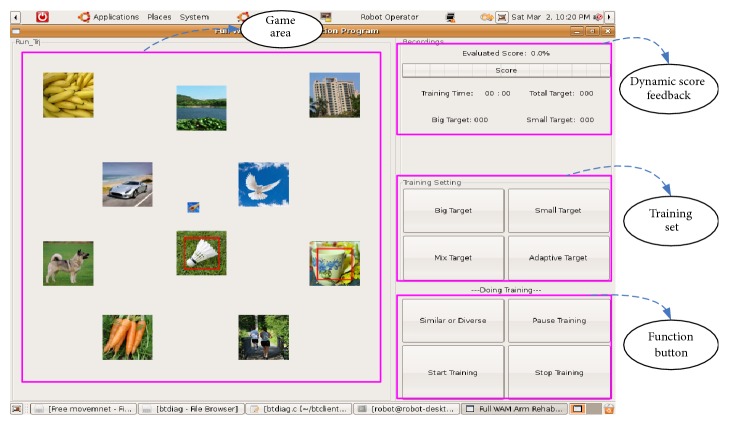
User interface for free movement training.

**Figure 3:**
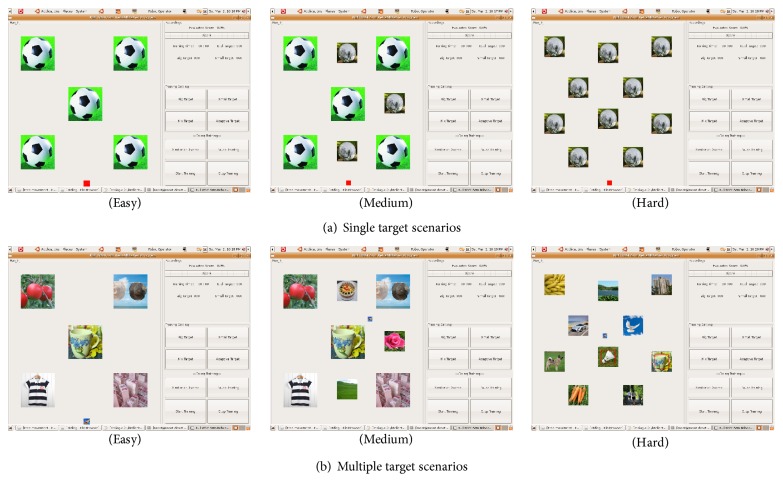
Different target scene modes.

The developed user interface (UI) for free movement training is shown in Figure 2, which mainly includes the game area, dynamic score feedback, training settings, function buttons, and other parts. The game area is used to present the target picture for free movement training. The dynamic score feedback section online displays the training score. The training setting section is used to select the difficulty level or mode, and the function button mainly includes the target scene mode and related game control buttons.  Figure 3 shows the specific game interface for a single target scene and multiple target scenes.

### 2.4. Adaptive Strategy

The scene mode and difficulty level of free motion training can be set through the relevant interface buttons. When the supplied training task is too easy or difficult for the subject, it is easy to get bored or frustrated. It is very important to serve the subject with appropriate task level matching his current ability.

To supply the subject with suitable task intelligently during the training, an adaptive task level strategy for free motion training is proposed in this research, and the task level for training is determined by the subject performance according to proposed strategy, which makes the subject always served with appropriate task matching his ability and being active. Meanwhile, to effectively stimulate the patient's active movement awareness, a dynamic score calculation method is utilized to evaluate the training performance and visual feedback to the subject in real time.

Usually, the game only records static grades, such as the number of captured targets. To effectively inspire the sense of urgency for the subject during the training, the dynamic score calculation method is used. The number of total captured targets, large icon targets, small icon targets, and the training time are recorded in the training. The training score is dynamically evaluated based on the training time and number of captured targets. If the subject does not capture any targets for a period of time, the evaluated score will gradually decrease, giving the subject a sense of urgency. During the training, when the moving “virtual hand” stays on the target for 2 s, it means that the target is captured, and then the target disappears on the screen. Considering that the quests vary in difficulty for grasping large icons and small icons, a convert coefficient is adopted when calculating the dynamic score. The dynamic scores are calculated by using the following function:

(1) $$\begin{array}{c} score=k_{a}\times 4\times \frac{k_{t}\left(m+k_{d}\times n\right)}{T} \end{array}$$

where *k*_*a*_, *k*_*t*_, *k*_*d*_, *m*, *n*, and *T* present adjustment ratio coefficients, grade factors, conversion coefficients, number of large icons, number of small icons, and time, respectively.

To avoid the influence of accidental factors when using the adaptive difficulty level mode training, the task difficulty level is adjusted based on the evaluated results of the previous three-unit performances. It is reasonable to use three-unit performances to evaluate and adjust the task difficulty, which avoids the limitations, being susceptible to accidental factors for single-unit evaluation with poor sensitivity for multiunit evaluation. Moreover, if the difficulty level is too high and completing the current unit training is difficult, the timeout mechanism is set. If the set time is exceeded, the patient will then be supplied with low-level training task. The adaptive strategy for adjusting the task level is as follows.

(a) If the mean score (at the end of each training) of the three-unit training is <80%, the task level of the next three-unit training will be downgraded. However, if the mean score is >90%, the training level will be increased. In other cases, the training task level remains the same.

(b) During the training, if no target is captured within continuous 45 s and the current dynamic assessment score is <60%, the task level is automatically degraded.

(c) If the mean score of the three-unit training is still <60%, and the patient is training with the lowest task level, the system factor *k*_*a*_ is increased to a certain value. If the patient is training with the highest task level, and the mean score of the three-unit score is >95%, the system factor *k*_*a*_ is reduced to a certain value.

With the aforementioned design adjustment rules, the system can adaptively provide appropriate free movement training for the patient, while enhancing training enthusiasm. Considerations, such as intuition, grabbing of the object, and dynamic score feedback, make the training movement more interesting and incentive based.

### 2.5. Participants Selection

For clinical experiments, the case screening criteria are as follows.

*Inclusion Criteria*. The standard of screening case is in accordance with the diagnostic standard adopted by the 4th Academic Conference of Cerebrovascular Disease in 1995. For participating in the clinical rehabilitation experiment, the patients must be affected with limb movement dysfunction with no serious cognitive impairment and mental illness, and their vital signs are also stable. Meanwhile, they must comprehend the experimental requirements and understand the experimental process and purpose and agree to participate in robot-aided motion rehabilitation training. 

*Exclusion Criteria*. Patients with mental illness before contracting a disease, demented performance before cerebral apoplexy, severe cognitive impairment and aphasia, or severe diseases of vital organs, such as heart, lung, liver, and kidney, neuromuscular lesion affecting functional recovery, and not cooperating with the doctor during the clinical rehabilitation training are excluded.

Based on the aforementioned inclusion-exclusion standards and considering the rehabilitation stage of the recruiter and continuity of the experiment, because the majority of hospitalized patients are in the early stages of recovery and discharge is usually chosen when being in free motion stage, two patients are selected for clinical trials from the 36 stroke in-patients.

## 3. Results and Discussion

### 3.1. Experimental Planning

**Figure 4:**
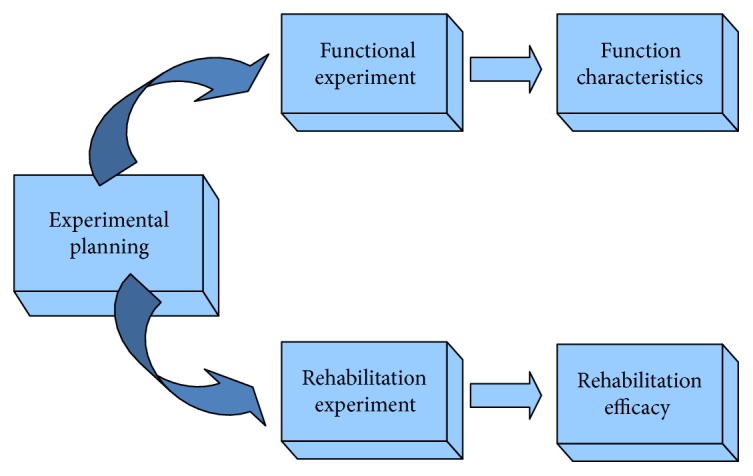
Experimental planning.

To further verify the characteristics and rehabilitation efficacy of the proposed adaptive strategy of training task level and dynamic score feedback for free movement training, the related experimental investigation was conducted. Experimental planning is shown in Figure 4. The function characteristics of the designed adaptive task level adjustment were presented and analyzed through a functional experiment with a healthy adult's movement training. Meanwhile, efficacy of the designed free movement training rehabilitation system was inspected by screening cases and conducting clinical trials.

### 3.2. Functional Experiments

To compare and analyze the advantages of the adaptive task level adjustment function, a healthy subject underwent free motion training under three different task levels and adaptive modes. For each set of experiments, each category training (easy level, medium level, hard level, adaptive mode) was continued for 10 min. To objectively and effectively analyze the performances of the designed rehabilitation system, five sets of experiments were conducted in 5 days, and the training categories in each training group were randomly arranged. Since the system function was indicated by experimental training of healthy adults, the experiment parameters *k*_*a*_ and *k*_*d*_ were set at values 0.6 and 1.3, respectively. The specific experimental results are shown in Table 1.

**Figure 5:**
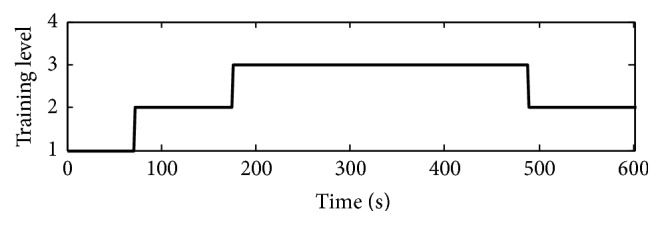
Training level adjustment for adaptive mode. Note: 1, 2, and 3 represent easy, medium, and hard levels, respectively.

From the results of Table 1, the grade changes slightly during the training with a single difficulty level mode. If the supplied task level does not match the subject, enhancing the patient's training enthusiasm is difficult. Moreover, the training task level cannot be adjusted based on the state of the subject during the training, and generating feelings of frustration or boredom for the executor is easy. Coming to the same motion training under adaptive mode, the range of training grade is quite wide (61%–100%), and the overall training grade is relatively stable (83.1%–88.5%). In the motion training with adapted mode, the task level is adjusted based on the training performances, which presents the subject with novelty and motivation and arouses the subject's challenge consciousness and sense of accomplishment. In addition, the adaptive mode is capable of intelligently adjusting the task level, demonstrating good adaptability, and can perform free movement training for patients with different illnesses.  Figure 5 shows the specific adjustment of the task level when training in adaptive mode.

### 3.3. Rehabilitation Experiments

**Figure 6:**
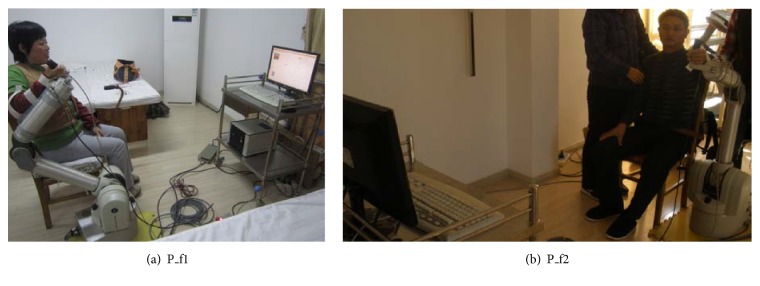
Clinical rehabilitation with free motion training.

The purpose of clinical rehabilitation training was to verify the efficacy of the proposed adaptive strategy with dynamic score feedback. After screening cases, two recruited stoke patients (P_f1, P_f2) performed free movement training on the designed motion rehabilitation system with adaptive mode.  Table 2 shows the information of two patients and Figure 6 presents the clinical rehabilitation scenario.

**Figure 7:**
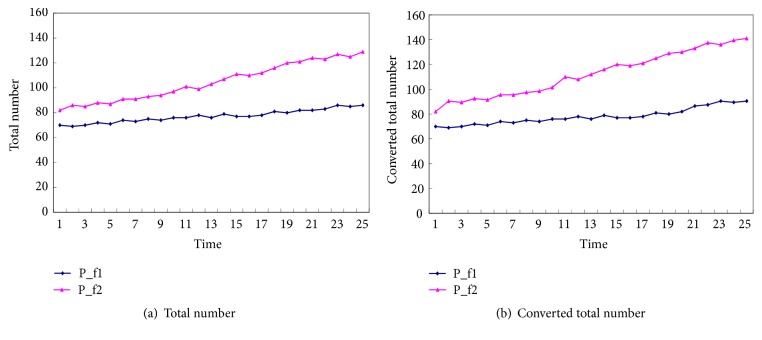
Change of grasping target number during the motion training.

**Figure 8:**
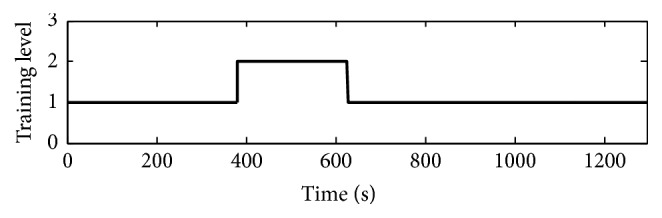
Training level adjustment for P_f2. Note: 1, 2, and 3 represent easy, medium, and hard levels, respectively.

The clinical rehabilitation training is conducted for five weeks (25 training days), and each subject is conducted to undergo one session with 20 min, one training day. During the 5 weeks training, the total number of captured goals and converted total number are shown in Figure 7, respectively. It can be acquired from Figure 7 that the total number of captured targets by the two patients is increasing during the training phase, which indicates that the movement control performances of the subjects are gradually improved. Comparing the total number of captured targets before and after training, it can be concluded that the movement flexibility and conformity of the affected limb have been improved with motion rehabilitation training, which is well demonstrated on P_f2 patient. The recorded adjustment of the task level for the patient's P_f2 in one session training is shown in Figure 8, which shows that the designed system can adaptively adjust the training task level based on the training performances during the training process and serve the subject with appropriate free movement training, which makes the motion training humanized and being what the subject wants.

### 3.4. Discussion

Currently, rehabilitation training robots are usually used to help patients complete specific motion training by providing auxiliary force, such as robot-aided passive, active, and resistant motion training. Few studies focus on robot-aided free motion training. In this study, a robot-aided free motion rehabilitation system was designed, and the adaptive task level strategy was proposed. Several training tasks based on the grabbing game with visual feedback were developed to make the training more attractive. The results of functional and clinical experiments suggest that the designed robot-aided free motion rehabilitation system with adaptive task level strategy and dynamic feedback was effective with respect to incentive function and rehabilitation efficacy. In the present investigation, we mainly verified the effectiveness of the proposed robot-aided free motion training mode and the adaptive task level and dynamic feedback strategy. In future studies, we will develop different training tasks and conduct additional clinical experimental investigations in a greater number of stroke patients and analyze the impact of training time and task type on treatment outcomes.

## 4. Conclusions

With the aim of clinical free motion training for stroke patients, an adaptive task level strategy was proposed in this study, which allows the training system to present the patient with the task of the appropriate training level, in accordance with his movement ability; this would stimulate the patient's enthusiasm for training to the maximum extent. Additionally, multiple training tasks based on visual feedback were developed, and the motion rehabilitation system was constructed for free motion training. To further enhance the patient's awareness of the training, the dynamic score feedback method was used while designing the training tasks. Functional experiments and clinical rehabilitation experiments were conducted to verify the functional characteristics and efficacy. The experimental results suggest that the proposed adaptive task level strategy, which intelligently supplies the subject with a suitable training task based on his previous performances, provides effective incentive function and rehabilitation efficacy.

## Figures and Tables

**Table 1:** Training results with different modes (%).

Group code	Easy level		Medium level		Hard level		Adaptive mode	
	Score range	Single mean score	Score range	Single mean score	Score range	Single mean score	Score range	Single mean score
1	87~100	93.4	77~89	81.4	62~85	68.5	61~100	83.1
2	83~100	89.1	80~93	87.3	59~78	65.1	66~100	88.5
3	87~100	94.8	81~90	84.6	64~86	72.7	63~100	86.1
4	86~100	93.7	75~88	80.1	59~82	67.3	63~100	84.6
5	84~100	91.5	79~92	83.8	61~83	70.4	64~100	84.3
Total mean	92.5		83.44		68.8		85.3	

**Table 2:** Information of the stroke patients.

Patient code	Age (years)	Gender	Time since stroke (months)	Impaired limb
P_f1	45	Female	6	Right
P_f2	47	Male	8	Left

## Data Availability

The data used to support the findings of this study are included within the article.
